# Selection of heat treatment conditions and prevention of secondary microbial contamination of liquid sugar: practical remarks

**DOI:** 10.1007/s13197-021-04971-3

**Published:** 2021-01-20

**Authors:** Ilona Błaszczyk, Jan Iciek

**Affiliations:** Faculty of Biotechnology and Food Sciences, Institute of Food Technology and Analysis, Lodz University of Technology, Lodz, Poland

**Keywords:** Sugar beet, Liquid sugar, Heat treatment, Prevention of secondary microbial contamination, Practical remarks

## Abstract

The purpose of the paper is to provide practical information on the selection of heat treatment conditions and the prevention of secondary microbial contamination of liquid sugar. The guidelines included in the paper were formulated on the basis of practical experience gained. The most important aspect often refers to the unnoticed cause of secondary contamination of liquid sugar after heat treatment, during its cooling in diaphragm heat exchangers along with the indication of the possibility of elimination of this cause. The article also presents the results of microbiological analysis in the field of heat resistance of microorganisms present in raw juice, obtained from the extractor. The data indicate the difficulty of thermal inactivation of mesophilic and thermophilic spores present in the studied environment. The cited results of microbiological analysis prove the usefulness of the measures to prevent secondary microbial contamination of the finished product.

## Introduction

Obtaining the desired microbiological status of finished food products is one of the most important goals for enterprises dealing with food processing. Enterprises in the food industry are supposed to obtain a finished product with the best possible microbiological quality. Microorganisms present in a finished food product intended for sale can multiply and change both its organoleptic and physicochemical characteristics as well as have a negative impact on its usefulness for consumption. Microorganisms that cause disease in humans may not be present in food. Food safety management is supported by various documents, including primarily legal acts. The legal documents that provide specific microbiological or chemical requirements for ready-made food products are particularly useful. The microbiological criteria for foods in Commission Regulation (EC) No 2073/2005 (European Commission 2005) apply to 'high-risk food'. This includes ready-to-eat foods for infants as well as for special medical purposes such as meat and meat products, milk and milk products, egg products, fishery products as well as vegetables, fruits and derivatives. The document specifies acceptable limits for the content of pathogenic bacteria such as: *Listeria monocytogenes, Salmonella, Cronobacter spp., Escherichia coli, and coagulase-positive staphylococci*. The above regulation does not contain microbiological criteria for sugar in either solid or liquid form. If there are no legal requirements for the microbiological status of the finished food product, the final criteria are based on the company's experience, customer requirements, normative documents or requirements developed by the associations. The microbiological criteria for liquid sugar refer to the total number of mesophilic bacteria, yeast and mold. The sample specification for liquid sugar determines the maximum amounts of microorganisms in colony forming units per 10 g of D.S.E (Dry Sugar Equivalent), which are for: mesophiles 200 CFU / 10 g of D.S.E, yeast 10 CFU / 10 g of D.S.E, mold 10 CFU / 10 g of D.S.E (Nordic Sugar Accessed 15 June 2019). The document does not specify requirements for thermophilic bacteria. However, the thermophilic spores present in sugar solutions arouse concern among manufacturers of some canned and non-alcoholic beverages. Thermophilic spores of aerobic bacteria (e.g., *Geobacillus stearothermophilus*) and anaerobic bacteria (e.g., *Clostridium thermosaccharolyticum*) have been associated with the spoilage of low-acid shelf-stable foods. These microorganisms are only problematic for shelf-stable foods that are distributed and stored at high ambient temperatures (International Commission on Microbiological Specifications for Foods 2011).

Therefore, the quality of the finished product is evaluated in terms of different groups of microorganisms. Temperature is one of the fundamental determinants the development of a specific group of microorganisms depends of. For example, due to the temperature of the sucrose extraction process carried out continuously, the mixture of beet cossettes with water is mainly inhabited by thermophilic bacteria (Poel et al. 1998).

The microbiological status of the finished product, e.g. liquid sugar, depends on the quality of the raw material and the technological conditions used as well as heat treatment (pasteurization, sterilization) during its production and the effectiveness of eliminating the causes of secondary microbiological contamination of the finished product. The conditions (temperature and time of operation) of heat treatment of liquid sugar depend on its microbiological state, i.e. the type and amount of microflora that colonizes it, which is the result of conditions prevailing both at the earlier stages of the technological process and between these stages. In order to achieve the assumed level of microbiological purity of the finished product in practice, it is necessary to get acquainted with different fields of knowledge such as: food technology and microbiology, unit processes, quality management, including food safety, engineering, in particular the construction and operation of equipment used for food production. Without such interdisciplinary knowledge it is difficult to find the right solutions to effectively prevent or minimize microbial contamination to an acceptable level, and yet we know that "prevention is better than cure". The microbiological aspect should be analyzed throughout the production chain for the possible multiplication of microorganisms and the effects of their activities, the effectiveness of inactivation of microorganisms and the elimination of the possibility of secondary contamination of the finished product. In the case of liquid sugar, the huge danger of secondary microbiological contamination occurs during its cooling in the diaphragm heat exchangers. Due to the high concentration of sucrose, liquid sugar is not an environment supporting multiplication of bacteria during its storage, but it is a convenient medium for multiplication of mold and yeast (Bergwall 2018). Secondary microbiological contamination of the finished product with a significant sucrose content such as liquid sugar is a huge problem for its producers. The elimination of microorganisms by heat treatment for this type of product requires the use of "acute" conditions.

The literature provides no information on the elimination of the cause of secondary microbial contamination, which results from improper operation of the apparatus.

The main purpose of this work is to obtain practical information on the prevention of secondary microbial contamination of liquid sugar.

## The principle of selecting the conditions of heat treatment of media during their pasteurization or sterilization

**Fig. 1:**
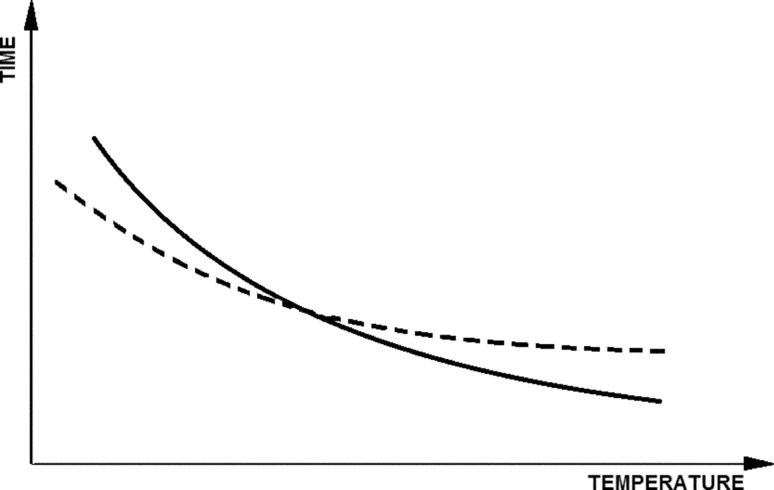
Illustration of the influence of heat treatment conditions of the media (pasteurization or sterilization) on the thermal degradation of components and inactivation of microorganisms

Current industrial practices reveal a tendency to use a higher temperature for the purpose of pasteurizing or sterilizing a medium achieving a short heat treatment time. In order to illustrate this principle, an illustrative drawing (Fig. 1) is presented regarding the selection of thermal treatment conditions for the media. The curves in the figure refer to a given medium with a specific chemical composition and microbiological state. These curves illustrate the general principle that applies both to the selection of pasteurization conditions and media sterilization. The course of curves depends on many parameters and therefore it must be strictly defined for each case, but the fundamental principle is similar. The required conditions for the effect of pasteurization or sterilization for a given medium are always represented by a continuous line.

This curve illustrates the relationship between temperature and heat treatment time. As the temperature rises, the required heat treatment time is reduced. At the same time, heat supplied to the medium during its heat treatment affects not only the microorganisms present in it, but also its chemical and physical properties. The practical experience demonstrates that in most cases heat treatment affects the properties of the medium with a specific tendency. This effect is illustrated by the dashed line specifying acceptable conditions for heat treatment of the medium, which will not cause a significant change in its properties in relation to the original state (before the process).

In practice, in order to guarantee the effectiveness of sterilization or pasteurization, a longer operating time of a given temperature (i.e. above a continuous line) is used than this required for the thermal inactivation of certain forms of microorganisms present in a given medium. At the same time, in order not to allow for significant chemical changes to occur in the medium due to the effect of elevated temperature, a time shorter than that illustrated by the dashed line should be applied. When analyzing the drawing (Fig. 1), it is clearly visible that using low temperatures for pasteurizing or sterilizing the medium will result in significant changes in the product properties. On the other hand, using a high temperature, one can choose the time from the area between these curves and obtain a product that has been pasteurized or sterilized, while not significantly altering its chemical properties.

In summary, figure (Fig. 1) illustrates the change in the relationship between curves, which represent respectively: continuous curve—effective thermal treatment allowing inactivation of certain forms of microorganisms depending on temperature, and dashed curve—acceptable conditions for thermal treatment of the medium due to the preservation of its chemical properties. The course of the curves shows that a higher temperature of pasteurization or sterilization allows for the effective inactivation of certain forms of microorganisms and at the same time it allows for good preservation of the properties of the medium.

## Selection of conditions for the pasteurization of liquid sugar

Depending on its intended use, liquid sugar may be pasteurized or sterilized. Liquid sugar which is a raw material for the pharmaceutical industry must be sterilized, i.e. all forms of microorganisms must be eliminated. Pasteurized liquid sugar is used mainly in the soft drinks industry, ice cream and dairy products. Liquid sugar is used for products such as marmalade, jam, pickled vegetables, sauces, ketchup and mustard. It can also be used as substrate in industrial fermentation (Nordic Sugar Accessed 15 June 2019).

**Fig. 2:**
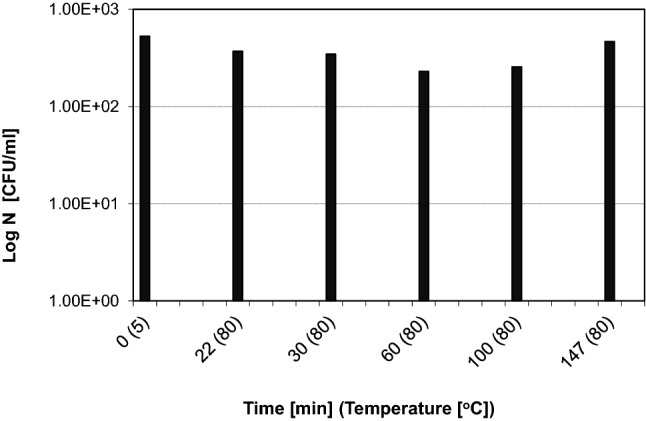
Number of spores of thermophilic bacteria present in raw juice (taken from the extractor) during its storage at temperature of 80 °C

Our research concerned the pasteurization of raw juice obtained from the extractor during the campaign from a sugar factory located in Poland. The study analyzed its microbiological status, i.e. mesophilic and thermophilic spore counts at different time of its storage at a pasteurization temperature of 80 °C. After heating the juice to the assumed temperature, it was kept at this temperature for two hours. Two substrates were used to determine the number of selected bacterial groups: PCA substrate for mesophilic spores and Cameron substrate for spores of thermophilic bacteria. The PCA substrate contained: yeast extract, peptone K, glucose, agar. Cameron's substrate consisted of: peptone K, glucose, bromcresol purple, agar. The results of own laboratory tests in the field of heat resistance of microorganisms colonizing the raw juice are presented in diagram (Fig. 2).

The obtained results indicate a significant heat resistance of the population of spors of mesophilic bacteria present in the raw juice collected from the extractor, because the long-term action of the temperature of 80 °C did not allow for their elimination. From the data obtained, it result that the population of spors of mesophilic bacteria colonizing the raw juice was resistant to the tested pasteurization temperature. The number of mesophilic bacterial spors remained at 10^2^ CFU / ml after 125 min of heating raw juice at 80 °C. It should be noted that the number of spors of mesophilic bacteria that was obtained after about 10 min after reaching the assumed temperature was maintained during further heating (for about 100 min). Thus, prolonging the pasteurization time did not allow for inactivation of mesophilic and thermophilic spores present in the raw juice. Based on data obtained during the microbiological analysis of raw juice during its long-term storage at 80 °C, the number of the maintained thermophilic spores was at the level of 10^2^ CFU / mL.

It should be emphasized that the obtained results may indicate not only a very high resistance of microorganisms present in the raw juice, but also the protective influence of environmental components on them. The author (Ołtuszak-Walczak 2002) kept the *Geobacillus stearothermophilus* spores in the temperature range 115 °C–125 °C in aqueous solutions of glucose, sucrose, starch, casein, peptone, molasses and milk powder. She stated that the tested substances have a protective effect on the survival of spores, extending the time necessary to carry out effective sterilization. Another author (Chmal-Fudali 2013) also found a significant extension of the thermal process of inactivation of *Geobacillus stearothermophilus* spores suspended in aqueous sucrose solutions (10% and 15%) at pH 5.0 and 7.0 compared to distilled water.

Inactivation of spors of mesophilic bacteria living in liquid sugar will be more difficult than their inactivation in raw juice resulting from the much higher sucrose content in liquid sugar. The presented results of microbiological tests show that the temperature of 80 °C is absolutely insufficient to inactivate mesophilic and thermophilic bacterial spores but sufficient to carry out effective pasteurization allowing to inactivate mesophilic and thermophilic vegetative forms. The requirements of the liquid sugar recipient must still be taken into account. The sample specification for liquid sugar does not specify requirements for spors (Nordic Sugar Accessed 15 June 2019). Practical experience shows that the process of pasteurization of liquid sugar is effective at 110 °C during the period of 5 s. According to the authors, these conditions will not have a negative impact on the properties of liquid sugar.

While analyzing the obtained results, one cannot omit the possibility of activating spores at this temperature, which can be confirmed by a slight increase in the number of spores of thermophilic bacteria at the end of the heat treatment process (Fig. 2, last column). The author (Molska 2002) showed that already under the conditions of pasteurization (80 °C, 10 min) the spores of *Geobacillus stearothermophilus* contained in distilled water with the addition of NaCl were activated. On the other hand, Błaszczyk (2007) found a clear activation of spores of *Geobacillus stearothermophlius* suspended in a tryptone solution (no acid added, and in the presence of citric acid with pH 6.00) after 3 min at 115 °C and after 20 s at 125 °C. Significant activation of *Geobacillus stearothermophilus* spores was also observed in real environments such as skimmed milk and asparagus juice, i.e. after 30 s at 121 °C. In the conditions cited, the number of germinable spores increased by approximately one order of magnitude.

## Prevention of secondary microbial contamination of pasteurized liquid sugar during its cooling

The causes of secondary microbiological contamination of the finished product may be of different character. Most of them are well-known. The secondary microbiological contamination of the finished food product may be caused by packaging material, leakage of the packaging, surface of devices used in the final stages of production, contact with non-sterile air or errors in operating the installation. The measures to prevent secondary microbial contamination include, among others, the preparation of effective procedures for cleaning and disinfecting equipment surfaces and their handling, selection and handling of packaging materials, and effective training for employees (Pierson and Corlett 1992, The European Parliament and the Council 2004). In order to obtain a finished product with the desired microbiological status, it is very important to know the construction and principles of operation of devices used for the implementation of technological unit processes. Improper handling may contribute to the secondary microbiological contamination of the finished product and all previous efforts to obtain its assumed microbiological purity can be wasted. Food manufacturers often have problems with the occurrence of non-systematic secondary microbiological contamination of the finished product, which results from improper device operation. The cause of this type of microbiological contamination of the product is very difficult to diagnose. The process of cooling of pasteurized liquid sugar in diaphragm heat exchangers using non-sterile media is an example of such problem related to the operation of the device.

Cooling the pasteurized medium in diaphragm heat exchangers using non-sterile media allows for a significant heat recovery. The recovery of heat from pasteurized liquid sugar is important from an economic and environmental point of view. This heat is used to preheat non-sterile liquid sugar or raw materials for its production. For this purpose, as well as for the final cooling of pasteurized liquid sugar, plate exchangers are used because of their efficiency of exchanging heat and a relatively low price. Unfortunately, there is no guarantee that these diaphragm heat exchangers are completely tight. In practice, there is a risk that the minimum amount of one medium can flow to the other through minimal damage to the seal of the plates separating both media (pasteurized and unpasteurized). The relatively minute mass exchange between these heat exchanging media is not of energetic and mass significance (for total heat streams), but it is of great importance for microbiological reasons. The minimum amount of non-sterile liquid sugar or cooling water passing into pasteurized liquid sugar causes its microbial secondary contamination. To eliminate this type of danger, the overpressure must be maintained on the side of the pasteurized liquid sugar in relation to the pressure in unpasteurized liquid sugar and cooling water.

**Fig. 3:**
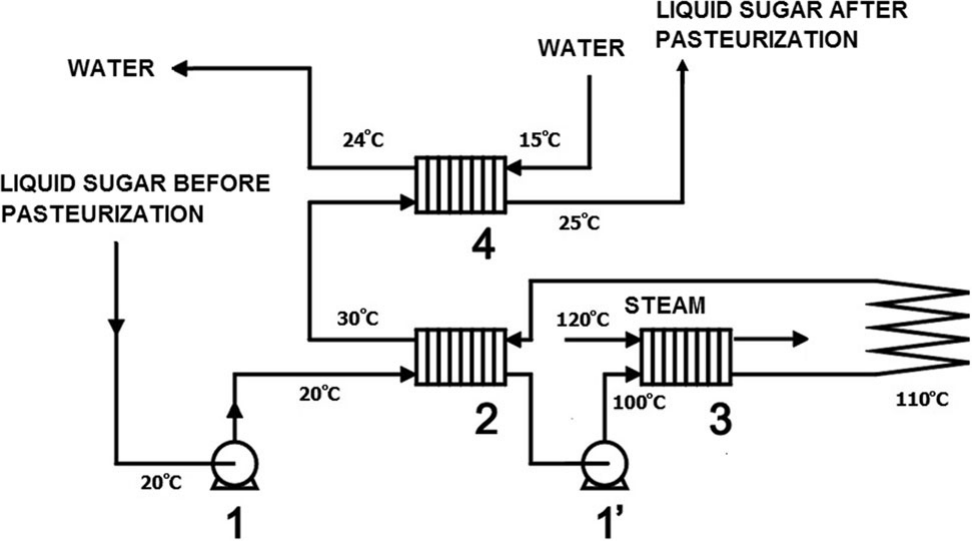
Diagram of the installation for pasteurization of liquid sugar and its cooling. Continuous curve—required conditions for thermal inactivation of microorganisms, Dashed curve—acceptable conditions for thermal treatment of the medium (for mantaining properties of the product). 1 and 1’ - pumps, 2, 3, 4 - diaphragm heat exchangers

The described cause of secondary microbial contamination of liquid sugar after the pasteurization process during its cooling in diaphragm heat exchangers can be effectively eliminated by using an additional pump (marked in Fig. 3 as 1′) and appropriate valves allowing the required pressure control. It allows to obtain overpressure in the pasteurized medium in relation to the unpasteurized medium. Figure 3 presents a solution allowing to eliminate the cause of secondary microbial contamination of liquid sugar during its cooling in diaphragm heat exchangers marked as 3 and 5. The figure also includes temperature values at the pasteurization stage and subsequent cooling of pasteurized liquid sugar.

## Conclusion

1. In order to effectively eliminate the vegetative forms of mesophiles present in liquid sugar and preserve its properties, it is recommended to pasteurize it at 110 ° C and within 5 s.
2. Based on our own research, it was found that mesophilic and thermophilic spores present in raw juice are resistant to a pasteurization temperature of 80 °C. Two-hour pasteurization of raw juice sourced from the extractor at 80 °C did not allow for inactivation of mesophilic and thermophilic spores present in it.
3. The use of an additional pump and appropriate valves to maintain the overpressure on the side of pasteurized liquid sugar in relation to unpasteurized liquid sugar and cooling water is an effective way to prevent the microbial contamination of pasteurized liquid sugar during its cooling in diaphragm heat exchangers using non-sterile media.
